# BK Channels in Tail Artery Vascular Smooth Muscle Cells of Normotensive (WKY) and Hypertensive (SHR) Rats Possess Similar Calcium Sensitivity But Different Responses to the Vasodilator Iloprost

**DOI:** 10.3390/ijms25137140

**Published:** 2024-06-28

**Authors:** Anastasia Pyanova, Vladimir N. Serebryakov, Hristo Gagov, Mitko Mladenov, Rudolf Schubert

**Affiliations:** 1 Physiology, Institute of Theoretical Medicine, Faculty of Medicine, University of Augsburg, 86159 Augsburg, Germany; anastasia.pyanova@med.uni-augsburg.de; 2 Institute of Experimental Cardiology, Cardiology Research Center, 121552 Moscow, Russia; 3 Department of Animal and Human Physiology, Faculty of Biology, Sofia University “St. Kliment Ohridski”, 1164 Sofia, Bulgaria; hgagov@uni-sofia.bg; 4 Institute of Biology, Faculty of Natural Sciences and Mathematics, University of Ss. Cyril and Methodius, 1000 Skopje, North Macedonia; m.mitko@gmail.com; 5 Department of Fundamental and Applied Physiology, Russian States Medical University, 117997 Moscow, Russia

**Keywords:** arteries, smooth muscle, calcium activated potassium channel, iloprost, spontaneously hypertensive rat

## Abstract

It has been reported that, in the spontaneously hypertensive rat (SHR) model of hypertension, different components of the G-protein/adenylate cyclase (AC)/Calcium-activated potassium channel of high conductance (BK) channel signaling pathway are altered differently. In the upstream part of the pathway (G-protein/AC), a comparatively low efficacy has been established, whereas downstream BK currents seem to be increased. Thus, the overall performance of this signaling pathway in SHR is elusive. For a better understanding, we focused on one aspect, the direct targeting of the BK channel by the G-protein/AC pathway and tested the hypothesis that the comparatively low AC pathway efficacy in SHR results in a reduced agonist-induced stimulation of BK currents. This hypothesis was investigated using freshly isolated smooth muscle cells from WKY and SHR rat tail artery and the patch-clamp technique. It was observed that: (1) single BK channels have similar current–voltage relationships, voltage-dependence and calcium sensitivity; (2) BK currents in cells with a strong buffering of the BK channel activator calcium have similar current–voltage relationships; (3) the iloprost-induced concentration-dependent increase of the BK current is larger in WKY compared to SHR; (4) the effects of activators of the PKA pathway, the catalytic subunit of PKA and the potent and selective cAMP-analogue Sp-5,6-DCl-cBIMPS on BK currents are similar. Thus, our data suggest that the lower iloprost-induced stimulation of the BK current in freshly isolated rat tail artery smooth muscle cells from SHR compared with WKY is due to the lower efficacy of upstream elements of the G-Protein/AC/BK channel pathway.

## 1. Introduction

Blood pressure and blood flow distribution to different organs depends to a large extent on peripheral vascular resistance, i.e., vascular tone of small arteries and arterioles. Vascular tone regulation is altered in a number of diseases, like hypertension, diabetes, and obesity (for an overview see [1]). Thus, in hypertension, in particular in the spontaneously hypertensive rat (SHR) model, a selective reduction of vasodilation induced by a variety of adenylate cyclase (AC) coupled agonists was observed in several arteries, which has been attributed to a reduced functional activity of the G-protein/AC pathway [2,3,4,5,6,7,8,9,10,11,12]. These alterations in vessel function in SHR are more unambiguously established for conduit arteries, but for smaller arteries the existence of these alterations and their possible mechanisms are still under debate [8,9,12,13,14]. Interestingly, the sensitivity of the blood pressure response to isoprenaline in vivo was reduced in SHR [12]. Furthermore, a reduced activation of AC has been proposed to be the cause for the smaller β-receptor mediated smooth muscle cell hyperpolarization in yet another hypertensive model, the reduced renal mass hypertensive rat, compared to normotensive rats [15].

The G-protein/AC signaling pathway in vascular smooth muscle cells (VSMCs) has a number of targets, in particular calcium-activated potassium channels of high conductance (BK channels) [16]. This channel is of special interest, as pioneering studies using the SHR model showed that inhibition of BK channels in different types of arteries (e.g., aorta; carotid, femoral, mesenteric, and cerebral arteries) revealed a larger functional impact of this channel on vascular contractility in hypertensive compared to normotensive rats (Wistar Kyoto rats, WKY) [17,18,19,20,21,22,23,24,25,26,27]. Consequently, this channel was suggested to contribute to altered contractile vessel function in this hypertension model, possibly as a mechanism limiting arterial contractions [28,29]. Of note, a smaller response to inhibition of BK channels has also been described in another hypertension model, suggesting that BK channels might contribute there to vasoconstriction and that their role may depend on the hypertension model studied [30].

In order to understand to what extent altered properties of the BK channel may contribute to its larger functional impact in the SHR model, BK currents in intact cells as well as single BK channel activity was studied. Thus, when the intracellular calcium concentration, the major activator of BK channels, was only weakly buffered at physiological concentrations, BK currents were larger in intact cells isolated from several arteries from SHR compared to WKY [17,20,21,23,26,27,31,32,33,34]. As this difference has not been observed in pre-hypertensive SHR and WKY, the genetic background seems not to be responsible for it [33]. In contrast, when the intracellular calcium concentration was below physiological values due to strong buffering or very low extracellular calcium concentration, BK currents were not different between SHR and WKY [32,35,36], suggesting that an enhanced activator calcium contributes considerably to the larger BK current in SHR [29]. Furthermore, single channel properties of the BK channel have been compared between cells isolated from several arteries of SHR and WKY. No differences have been observed in BK channel single channel conductance [20,26,28,30,36] and voltage-dependence [20,36]. However, contradictory findings have been reported regarding its calcium-sensitivity: a larger calcium-sensitivity of the BK channel in SHR has been reported in [26,27,28], but no difference was shown in [20,30,36,37].

Thus, it has been reported that, in the SHR model of hypertension, different components of the G-protein/AC/BK channel signaling pathway are altered differently. In the upstream part of the pathway (G-protein/AC), a comparatively low efficacy [2,3,4,5,6,7,8,9,10,11,12,14] has been established, whereas downstream BK currents seem to be increased [17,20,21,23,26,27,31,32,33,34]. However, the coupling between upstream G-protein/AC and the downstream BK channels is complex. Thus, the BK channel may be targeted directly by PKA acting on the BK channel [16] or indirectly by PKA affecting components regulating the local activator calcium for the BK channel, like voltage-gated calcium channels and calcium sparks. For example, PKA appears to stimulate voltage-gated calcium channels (see review for detailed discussion [38]) and may evoke persistent Ca^2+^ sparklet activity [39]. Further, this pathway has been shown to stimulate calcium sparks by increasing spark frequency [40], partly via phospholamban phosphorylation and disinhibition of the SR calcium pump [41]. Importantly, these components regulating the local activator calcium for the BK channel may also be different in SHR compared to WKY. Thus, in SHR, larger voltage-gated calcium currents [42,43,44] and increased density of Ca^2+^ sparklet sites associated with larger calcium currents [45] have been observed. Regarding spontaneous calcium sparks, a higher amplitude in SHR with similar frequency [27] as well as no differences in amplitude and frequency [46], have been reported. These data show that the G-protein/AC/BK channel signaling pathway is complex, with components affected differently in SHR. Thus, the overall performance of this signaling pathway in SHR is elusive. For a better understanding, we focused on one aspect, the direct targeting of the BK channel by the G-protein/AC pathway and tested the hypothesis that the comparatively low adenylate-cyclase pathway efficacy in SHR results in a reduced agonist induced stimulation of BK currents.

## 2. Results

### 2.1. Comparison of the Properties of BK Channels in Tail Artery VSMCs of WKY and SHR

**Figure 1:**
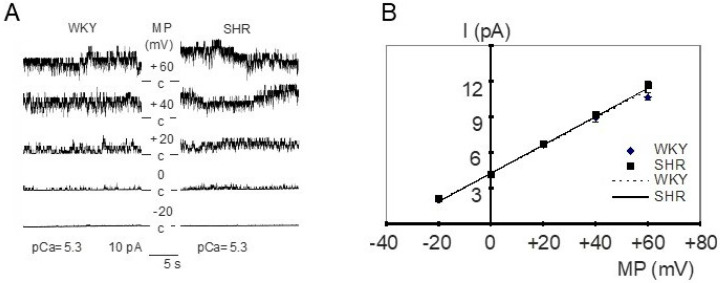
(**A**) Examples of BK channel activity in inside-out patches from WKY (left) and SHR (right) tail artery smooth muscle cells. The membrane potential (MP) was varied from −20 mV to +60 mV at a fixed pCa of 5.3 and an increase of channel amplitude and channel activity is observed upon depolarization. c = closed level of the channel, channel openings are shown as upward deflections. (**B**) Dependence of single BK channel amplitude (I) on membrane potential (MP) in inside-out patches excised from tail artery smooth muscle cells of WKY and SHR at pCa 5.3 (*n* = 7,6; *p* = 0.27; repeated measures ANOVA).

**Figure 2:**
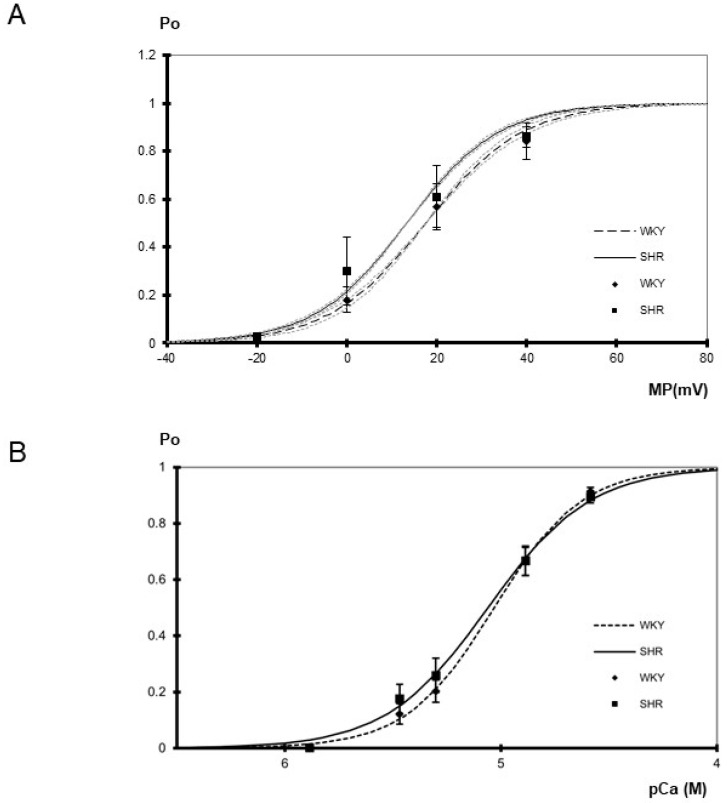
(**A**) Dependence of BK channel open probability (Po) on membrane potential (MP) in inside-out patches excised from tail artery smooth muscle cells of WKY and SHR (*n* = 4, 4; *p* = 0.91; two-way ANOVA) at pCa 5.3. Channel open probability increased upon depolarization (*p* < 0.05; two-way ANOVA). The gray dotted curves show the variability of the slope of the respective Boltzmann curves. They were obtained using the same membrane potential at which open probability reached 0.5 as for the original Boltzmann curve but using a slope increased and decreased, respectively, by its s.e.m. (**B**) Dependence of BK channel open probability (Po) on the intracellular calcium concentration in inside-out patches excised from tail artery smooth muscle cells of WKY and SHR (*n* = 8–10; *p* = 0.57, two-way ANOVA) at 0 mV. Channel open probability was larger at higher levels of the intracellular calcium concentration (*p* < 0.05; two-way ANOVA). A fit of a Hill function to the mean open probabilities at different calcium concentrations revealed similar slopes of 2.14 in WKY and 1.84 in SHR, as well as similar pD_2_ values of 5.03 in WKY and 5.06 in SHR.

In inside-out patches from WKY and SHR tail artery smooth muscle cells, the main channel activity had the following characteristics (see Figure 1A for examples). Channel openings possessed a large amplitude, which increased after membrane depolarization from −20 mV to +60 mV (see also Figure 1B). Further, this membrane depolarization resulted in higher channel activity characterized by longer and more frequent channel openings and a larger number of simultaneously open channels (Figure 1A). Additionally, channel activity increased remarkably after the application of a high calcium solution (see also Figure 2B). Furthermore, in outside-out patches, this channel was blocked by 1 mmol/L tetraethylammonium, which produced frequent transitions between the open and the closed state of the channel during channel open events resulting in a reduction of channel amplitude, and by 100 nmol/L iberiotoxin, which produced long lasting closed events without altering the channel amplitude (see previous reports [47,48]). Altogether, these properties indicate that the main channel activity in these patches belongs to the large conductance calcium-activated potassium channel (BK channel).

The current–voltage relationship of single BK channels from WKY and SHR tail artery smooth muscle cells was determined in the membrane potential range from −20 mV to +60 mV. Under our experimental conditions, a linear current–voltage relationship was observed. We did not detect any relevant difference between WKY and SHR (Figure 1B); in particular, single BK channel conductance was 116.8 ± 1.2 pS (*n* = 7) in WKY and 118.5 ± 5.6 pS (*n* = 6) in SHR (*p* = 0.76; *t*-test). Additionally, we did not detect any relevant difference between WKY and SHR regarding the position of the current–voltage relationship relative to the voltage-axis, as the single channel amplitude at 0 mV was 4.3 ± 0.1 pA (*n* = 7) in WKY and 4.4 ± 0.2 pA (*n* = 6) in SHR (*p* = 0.78; *t*-test).

Furthermore, the voltage-dependence of single BK channels from WKY and SHR tail artery smooth muscle cells was determined in the membrane potential range from −20 mV to +40 mV at pCa 5.3. A steep voltage-dependence was observed. We did not detect any relevant difference between WKY and SHR (Figure 2A). Thus, a fit of the dependence of single BK channel open probability on membrane potential with a Boltzmann function revealed a voltage dependence of 11 ± 1 mV (*n* = 4) change in membrane potential for an e-fold change in open probability in WKY and of 10 ± 1 mV (*n* = 4) change in membrane potential for an e-fold change in open probability in SHR (*p* = 0.65; *t*-test). The potential at which open probability reached 0.5 was 18 ± 4 mV in WKY (*n* = 4) and 13 ± 7 mV in SHR (*n* = 4) (*p* = 0.64; *t*-test). Additional data were obtained using only a one-step membrane potential change from 0 mV to +20 mV at several intracellular calcium concentrations. We did not detect any relevant difference between WKY and SHR (Table 1).

Finally, the calcium-sensitivity of single BK channels from WKY and SHR rat tail artery smooth muscle cells was determined in the range from pCa 5.9 to pCa 4.6 at 0 mV. Of note, each patch was exposed to only one calcium concentration. A prominent calcium-sensitivity was observed. We did not detect any relevant difference between WKY and SHR (Figure 2B). Further, the activity of the BK channel was measured at an additional membrane potential of +20 mV at a limited number of intracellular calcium concentrations. Thus, at pCa 5.3, channel open probability was 0.50 ± 0.07 (*n* = 8) in WKY and 0.54 ± 0.08 (*n* = 9) in SHR and at pCa 4.9 open probability was 0.85 ± 0.03 (*n* = 5) in WKY and 0.86 ± 0.02 (*n* = 5) in SHR, where we did not detect any relevant difference (*p* = 0.77 and *p* = 0.96, respectively).

### 2.2. Comparison of the Properties of BK Currents in Tail Artery VSMCs of WKY and SHR

**Figure 3:**
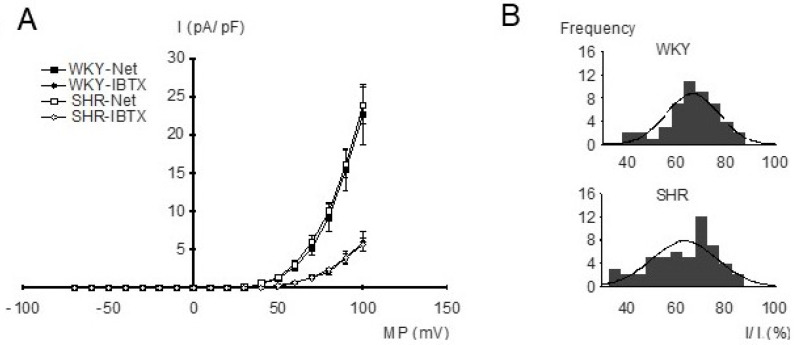
(**A**) Dependence of outward current amplitude on membrane potential in WKY and SHR tail artery smooth muscle cells. Current density (I), voltage (MP), relationship of the net outward current (squares) and the iberiotoxin (IBTX)-insensitive current (diamonds) from WKY and SHR (*n* = 11; *p* = 0.75 and *p* = 0.92, respectively; repeated-measures ANOVA). The BK current is the difference between the net outward and the iberiotoxin-insensitive current. (**B**) Amplitude histograms of normalized BK currents (normalized BK current = (net outward − iberiotoxin-insensitive current)/net outward current), expressed in percent. A Kolmogorov–Smirnov test demonstrated a normal distribution of BK current amplitudes; the solid lines show Gaussian curves fitted to the histograms.

For the investigation of the properties of BK currents in freshly isolated, intact tail artery smooth muscle cells from WKY and SHR, the whole-cell mode of the patch-clamp technique and strong buffering of the activator calcium for the BK channel were used. The current–voltage relationships were determined using voltage ramps from −70 mV to +100 mV based on a holding potential of −40 mV. Under our experimental conditions, an outward-rectifying current–voltage relationship was observed. We did not detect any relevant difference in the current–voltage relationships of the net outward current and of the iberiotoxin-insensitive current between WKY and SHR (Figure 3A), demonstrating similar BK currents (difference between net outward and iberiotoxin-insensitive current) in WKY and SHR. Indeed, histograms of normalized BK current amplitudes evoked by voltage steps from a holding potential of −40 mV to a test potential of +70 mV showed a normal distribution and we did not detect any relevant difference between WKY (mean 63.3 ± 1.8%; *n* = 53) and SHR (mean 66.1 ± 1.6%; *n* = 48) (*p* = 0.27) (Figure 3B).

### 2.3. Comparison of the Effect of Iloprost on BK Currents in Tail Artery VSMCs of WKY and SHR

**Figure 4:**
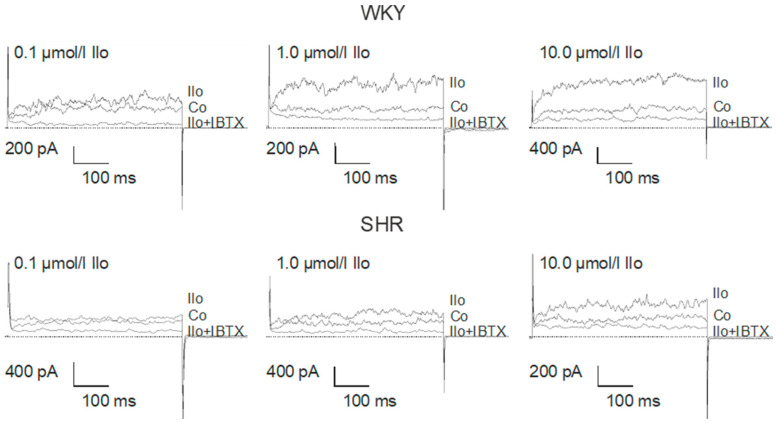
Examples of the effect of iloprost on the BK current of rat tail artery smooth muscle cells from WKY and SHR. Currents were evoked using 500 ms long pulses from a holding potential of −40 mV to a test potential of +70 mV. Only one concentration of iloprost was tested on each cell. Co, control current; Ilo, current after application of iloprost at the concentration shown; Ilo + IBTX, current after the subsequent addition of 100 nmol/L iberiotoxin.

Application of iloprost produced a concentration-dependent increase of the BK current in freshly isolated, intact tail artery smooth muscle cells from WKY (comparatively high AC pathway efficacy) and SHR (low AC pathway efficacy), which reached steady state 2–3 min. after the beginning of the application (Figure 4).

**Figure 5:**
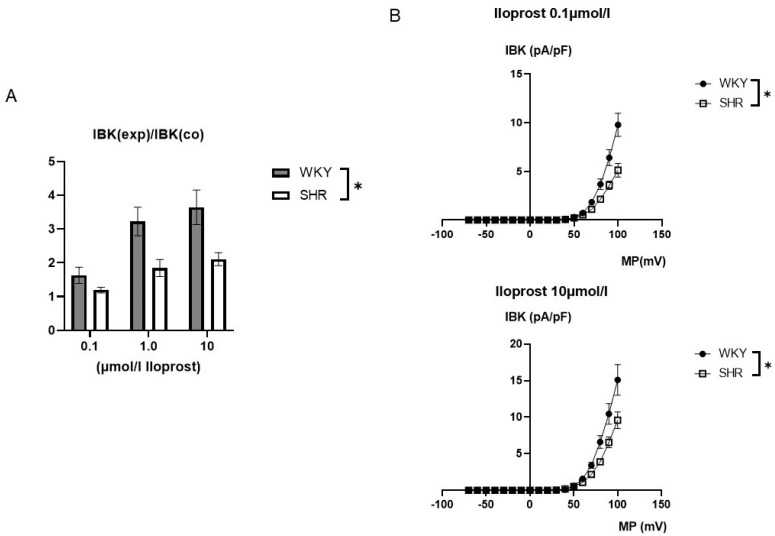
(**A**) Summary of the effect of iloprost on the BK current of rat tail artery smooth muscle cells from WKY and SHR. Currents were evoked using 500 ms long pulses from a holding potential of −40 mV to a test potential of +70 mV. The iloprost-induced increase of the BK current is expressed as the ratio of the BK current after application of iloprost (IBK(exp)) to the control current (IBK(co)). (**B**) Dependence of the iloprost-induced BK current density (IBK) on membrane potential (MP) in WKY and SHR tail artery smooth muscle cells. The BK current was normalized to the BK current at +70 mV before drug application. Upper panel: BK currents after application of 0.1 µmol/L iloprost Lower panel: BK currents after application of 10.0 µmol/L iloprost. * *p* < 0.05, repeated measures ANOVA.

The iloprost-induced increase in the BK current was larger in WKY compared to SHR (Figure 5). On average, 0.1 µmol/L iloprost increased the BK current at +70 mV 1.63 ± 0.24-fold (*n* = 10, *p* < 0.05, *t*-test) in WKY and 1.19 ± 0.08-fold (*n* = 11, *p* < 0.05; *t*-test) in SHR; 1.0 µmol/L iloprost increased the BK current 3.23 ± 0.42-fold (*n* = 12; *p* < 0.05; *t*-test) in WKY and 1.85 ± 0.25-fold (*n* = 12; *p* < 0.05; *t*-test) in SHR; and 10.0 µmol/L iloprost increased this BK current 3.65 ± 0.51-fold (*n* = 9; *p* < 0.05; *t*-test) in WKY and 2.11 ± 0.19-fold (*n* = 9; *p* < 0.05; *t*-test) in SHR; this effect was larger in WKY compared to SHR (*p* < 0.05; two-way ANOVA) (Figure 5A).

Additionally, exploring a wide range of membrane potentials using voltage ramps from −70 mV to +100 mV based on a holding potential of −40 mV, larger iloprost-induced BK currents were observed in WKY compared to SHR at 0.1 µmol/L iloprost (*n* = 9; *p* < 0.05; repeated measures ANOVA) and at 10.0 µmol/L iloprost (*n* = 9; *p* < 0.05; repeated measures ANOVA) (Figure 5B).

### 2.4. Comparison of the Effect of Activation of Components of the PKA Pathway on BK Currents in Tail Artery VSMCs of WKY and SHR

We have previously shown that the effect of iloprost on the BK current of rat tail artery smooth muscle cells is mediated by cAMP-dependent protein kinase (PKA) [47]. Thus, the effect of an activation of several components of the PKA signaling pathway on the BK current were studied in freshly isolated, intact tail artery smooth muscle cells from WKY (comparatively high AC pathway efficacy) and SHR (low AC pathway efficacy).

**Figure 6:**
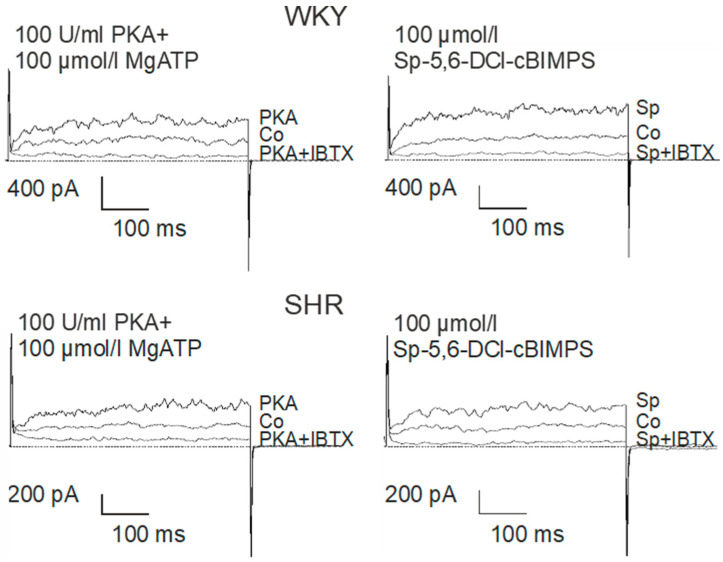
Examples of the effect of PKA (**left**) and Sp-5-6-DCl-cBIMPs (**right**) on the BK current of rat tail artery smooth muscle cells from WKY and SHR. Currents were evoked using 500 ms long pulses from a holding potential of −40 mV to a test potential of +70 mV. Only one AC/PKA-related agent was tested on each cell. Co, control current; PKA, current after application of the catalytic subunit of PKA together with MgATP; PKA + IBTX, current after the subsequent addition of 100 nmol/L iberiotoxin; Sp, current after application of the specific PKA-activator Sp-5,6-DCl-cBIMPS; Sp + IBTX, current after the subsequent addition of 100 nmol/L iberiotoxin.

The catalytic subunit of PKA at 100 U/mL together with 100 µmol/L MgATP were added to the pipette solution. About 1 min. after establishing the whole-cell configuration, the BK current started to rise in the presence of PKA and MgATP and after 5–6 min. a steady-state was obtained (Figure 6). Next, 100 µmol/L of the potent and selective cAMP-analogue Sp-5,6-DCl-cBIMPS, a PKA activator [49], was added to the pipette solution. About 1 min. after establishing the whole-cell configuration the BK current started to rise in the presence of Sp-5,6-DCl-cBIMPS and after 5–6 min. a steady-state was obtained (Figure 6).

**Figure 7:**
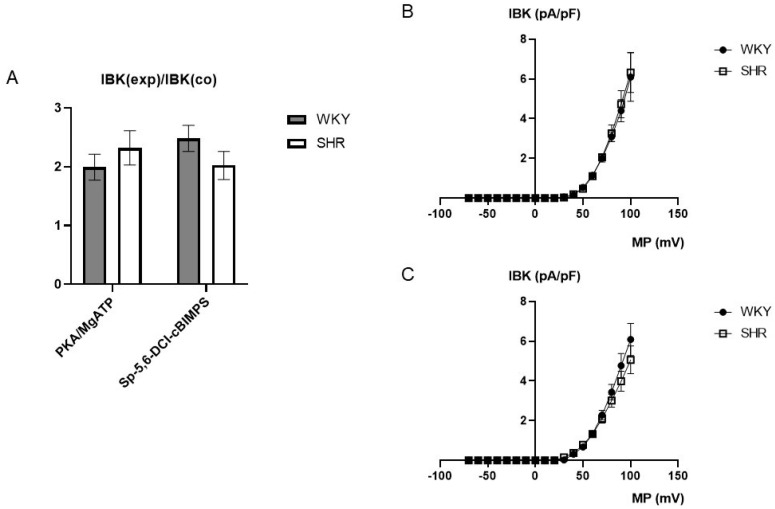
(**A**) Summary of the effect of 100 U/mL PKA together with 100 µmol/L MgATP as well as of 100 µmol/L Sp-5,6-DCl-cBIMPS on the BK current of rat tail artery smooth muscle cells from WKY and SHR. Currents were evoked using 500 ms long pulses from a holding potential of −40 mV to a test potential of +70 mV. The increase in the BK current is expressed as the ratio of the BK current after application of the substances (IBK(exp)) to the control current (IBK(co)). (**B**) Dependence of BK current density (IBK) induced by application of 100 U/mL PKA together with 100 µmol/L MgATP on membrane potential (MP) in WKY and SHR tail artery smooth muscle cells. The BK current was normalized to the BK current at +70 mV before drug application. (**C**) Dependence of BK current density (IBK) induced by application of 100 µmol/L Sp-5,6-DCl-cBIMPS on membrane potential (MP) in WKY and SHR tail artery smooth muscle cells. The BK current was normalized to the BK current at +70 mV before drug application.

On average, 100 U/mL PKA together with 100 µmol/L MgATP increased BK currents at +70 mV 1.99 ± 0.22-fold (*n* = 6; *p* < 0.05; *t*-test) in WKY and 2.32 ± 0.29-fold (*n* = 6; *p* < 0.05; *t*-test) in SHR (Figure 7A), but we did not detect any relevant difference between WKY and SHR (*p* = 0.41; *t*-test). Further, 100 µmol/L Sp-5,6-DCl-cBIMPS increased the BK current at +70 mV 2.48 ± 0.22-fold (*n* = 11; *p* < 0.05; *t*-test) in WKY and 2.02 ± 0.24-fold (*n* = 9; *p* < 0.05; *t*-test) in SHR (Figure 7A), but we did not detect any relevant difference between WKY and SHR (*p* = 0.19; *t*-test).

Additionally, exploring a wide range of membrane potentials using voltage ramps from −70 mV to +100 mV based on a holding potential of −40 mV, we did not detect any relevant differences between WKY and SHR after application of 100 U/mL PKA together with 100 µmol/L MgATP (*n* = 6; *p* = 0.83; repeated measures ANOVA) (Figure 7B) and of 100 µmol/L Sp-5,6-DCl-cBIMPS (*n* = 8; *p* = 0.49; repeated measures ANOVA) (Figure 7C).

## 3. Discussion

The main findings of the present study are: (1) single BK channels from WKY and SHR tail artery smooth muscle cells have similar current–voltage relationships, voltage-dependence and calcium-sensitivity; (2) BK currents in freshly isolated, intact cells with a strong buffering of the activator calcium for the BK channel have similar current–voltage relationships; (3) the iloprost-induced concentration-dependent increase of the BK current in freshly isolated, intact tail artery smooth muscle cells is larger in WKY compared to SHR; (4) the effects of activators of the PKA pathway, the catalytic subunit of PKA, as well as the potent and selective cAMP-analogue Sp-5,6-DCl-cBIMPS, on BK currents are similar.

### 3.1. Properties of BK Channels and BK Currents in Tail Artery VSMCs of WKY and SHR

Under the experimental conditions of the present study, i.e., with physiological, non-symmetrical distribution of potassium ions at the membrane, BK channel conductance was in the range between 115 and 120 pS for both WKY and SHR. This high conductance is typically observed for this channel in VSMCs. In particular, when the potassium ion distribution at the membrane was comparable to the conditions used in our study, similar conductance values of 125 pS [50] and 118 pS [51] have been reported. Of note, we could not detect any relevant difference in BK channel single channel conductance between WKY and SHR. Several previous studies have also demonstrated that the values of BK channel conductance (with symmetrical distribution of potassium ions at the membrane) do not deviate between WKY and SHR [20,26,28,30,36]. Regarding the latter observation, as far as we are aware, no contradictory results have been reported so far. Thus, our data strengthen the conclusion derived from previous studies that vascular smooth muscle BK channel conductance is similar in WKY and SHR.

Another characteristic property of the BK channel is its voltage-dependence. Voltage-dependence was analyzed by fitting the dependence of open probability on membrane potential with a Boltzmann function. The slope of this function, which represents the change in membrane potential required to get an e-fold alteration in open probability, was about 10–11 mV for both WKY and SHR. This slope is typically observed for this channel in VSMCs, where similar slopes of 11 mV [52], 11–13 mV [53], 15 mV [54], 16 mV [55], and 12–17 mV [20] have been reported. Of note, we could not detect any relevant difference in BK channel voltage-dependence between WKY and SHR. Previous studies have also demonstrated that BK channel voltage-dependence does not deviate between WKY and SHR [20,36]. Regarding the latter observation, as far as we are aware, no contradictory results have been reported so far. Thus, our data strengthen the conclusion derived from previous studies that vascular smooth muscle BK channel voltage-dependence is similar in WKY and SHR.

The most interesting functional property of the BK channel is its calcium-sensitivity. Calcium-sensitivity was analyzed by fitting the dependence of mean open probability on the calcium concentration with a Hill function. Under our experimental conditions, BK channel calcium-sensitivity at 0 mV was characterized by a pD_2_ in the range from 5 to 5.1 for both WKY and SHR. Data from the literature suggest that the calcium-sensitivity of vascular smooth muscle cell BK channels can vary considerably. Thus, at 0 mV a wide range of pD_2_s of e.g., 6.7 [56], 5 [55] and 4 (estimated from the data presented in [57]) has been reported. These differences in BK channel calcium-sensitivity can be explained to some extent by methodological differences between studies, especially when considering the numerous potential pitfalls involved in preparing buffer solutions with a given free calcium concentration (for details see chapter 7 in [58]). However, co-existence of two isoforms of the BK channel with respect to their calcium-sensitivity was reported in one and the same study, e.g., on bovine mesenteric artery smooth muscle cells with pD_2_s of 6.7 and 5 at 0 mV [56]. Differences in BK channel calcium-sensitivity were also found between different vascular beds, suggesting the presence of BK channel isoforms with different calcium-sensitivities [55]. Differences in calcium-sensitivity may be caused either by differential expression of splice variants of the pore-forming α-subunits, as suggested by [59,60], or by differences in the expression and/or coupling ratio of regulatory subunits, especially the β1-subunit, and pore-forming α-subunits [61,62,63]. With respect to the present study, inspection of the distribution of individual values of open probabilities at a given calcium concentration revealed no evidence for the existence of BK channel isoforms for both WKY and SHR. Of note, we could not detect any relevant difference in BK channel calcium-sensitivity between WKY and SHR, neither in terms of pD_2_ values nor in terms of another indicator of calcium-sensitivity derived from the voltage-dependence of the channels, namely the potential at which open probability reaches 0.5. Several previous studies have also demonstrated that BK channel calcium-sensitivity does not deviate between WKY and SHR [20,30,36,37]. Other studies have reported a larger calcium-sensitivity of the BK channel in SHR [26,27,28]. The observation that BK channel calcium-sensitivity in WKY and SHR appears to be different in some studies but similar in others may be due to the same reasons that explain general differences in BK channel calcium-sensitivity mentioned above. Thus, an increased protein expression ratio of the regulatory β1- and the pore-forming α-subunit was found in SHR compared to WKY in vessels in which BK channels with an increased BK channel calcium-sensitivity could be detected [25,26,27]. However, methodological issues cannot be excluded, but are rather unlikely, since the conclusions of similar or dissimilar BK channel calcium-sensitivity in WKY and SHR were drawn in one and the same study using the same methodological approach. To our knowledge, the role of splice variants of the BK channel α-subunit in hypertension has not yet been investigated. However, the exact reason(s) for these divergent findings on calcium-sensitivity of the BK channel between WKY and SHR is unclear and requires specific comparative studies in the future. Thus, our data extend the list of studies in which the calcium-sensitivity of the BK channel is similar in WKY and SHR.

To understand the role of BK channels in intact cells, BK currents were investigated in freshly isolated smooth muscle cells of the tail artery. BK currents have been shown to be activated by local calcium entry via voltage-gated calcium channels [64] and/or calcium release via calcium sparks, local calcium release events, which do not affect the global calcium concentration [65]. Importantly, in SHR compared to WKY, components that regulate the local activator calcium for the BK channel in intact cells were reported to be altered. For example, an increase of the amplitude of voltage-gated calcium currents [42,43,44], of the density of calcium sparklet sites, clusters of voltage-gated calcium channels [45] and of the amplitude of spontaneous calcium sparks [27] were observed in SHR. Since we focused on the direct control of the BK channel by the G-protein/AC pathway independent of its effects on the activator calcium for the BK channel, BK currents were studied using strong buffering of the activator calcium. Under these experimental conditions, we could not detect any relevant difference in BK currents in intact cells between WKY and SHR. Previous studies have also demonstrated that BK currents were not different between WKY and SHR when the intracellular calcium concentration was below physiological values due to strong buffering or very low extracellular calcium concentration [32,35,36]. Only when the intracellular calcium concentration was weakly buffered at physiological concentrations were BK currents larger in cells isolated from several arteries of SHR compared to WKY [17,20,21,23,26,27,31,32,33,34]. Thus, our data strengthen the conclusion derived from previous studies [29] that it is an enhanced activator calcium concentration that is required for a larger BK current in SHR.

### 3.2. Effect of Activation of Components of the PKA Pathway on BK Currents in Tail Artery VSMCs of WKY and SHR

It has been shown that PKA not only targets the BK channel directly [16], but also affects mechanisms that regulate the local activator calcium for the BK channel, such as calcium influx through voltage-gated calcium channels (see review for detailed discussion [38]), calcium sparklets [39] and calcium sparks [40,41]. Since we focused on the direct control of the BK channel by the G-protein/AC pathway independent of its effects on the activator calcium for the BK channel, BK currents were studied using strong buffering of the activator calcium. It has been shown that these experimental conditions greatly reduce the influence of mechanisms that regulate the intracellular calcium concentration on the BK current [66]. The activation of the G-protein/AC pathway was induced by iloprost. Previously, we had shown that iloprost stimulates BK currents in rat tail artery smooth muscle cells, an effect mediated by the AC pathway leading to vasodilation [47,67]. Under these experimental conditions, iloprost produced a concentration-dependent increase of the BK current in freshly isolated, intact tail artery smooth muscle cells also in the present study. A stimulation of BK currents by AC-coupled agonists is typically observed in vascular smooth muscle [1,16]. As demonstrated for the tail artery [47] and discussed in detail in [16,68], the molecular characteristics of the stimulation of the BK channel by the AC pathway (e.g., requirement for cAMP and MgATP, role of phosphatases, features of the interaction with PKA inhibitors) strongly suggest that phosphorylation of the channel underlies the PKA-induced stimulation of the BK channel. Indeed, a PKA-mediated phosphorylation at Ser 873 (in rat BK channels) has been reported to be responsible for BK channel activation [69,70]. Thus, AC-coupled agonists induce an increase in the intracellular cAMP concentration, cAMP binds to the regulatory subunits of PKA (PKA consists of 2 catalytic and 2 regulatory subunits [71]) leading to PKA activation and subsequent BK channel stimulation.

The effect of iloprost on BK currents was larger in WKY compared to SHR. Since WKY is characterized by a comparatively high AC pathway efficacy and SHR by a lower AC pathway efficacy [2,3,4,5,6,7,8,9,10,11,12,14], these data suggest that the difference in the iloprost-induced BK current between WKY and SHR seems to be due to a difference in the efficacy of iloprost in stimulating this current. Regarding the latter observation, as far as we are aware, similar data have not been reported so far.

In the SHR model of hypertension, the upstream part of the G-protein/AC pathway has been reported to be responsible for the lower pathway efficacy [2,3,4,5,6,7,8,9,10,11,12,14]. To test this idea in the present study, the effect of an activation of downstream components of the AC signaling pathway on the BK current was explored in freshly isolated, intact tail artery smooth muscle cells of WKY and SHR. The catalytic subunit of PKA together with MgATP, as well as the potent and selective cAMP-analogue Sp-5,6-DCl-cBIMPS, a PKA activator [49], applied via the patch pipette, increased the BK current. However, we could not detect any relevant difference in BK currents induced by either the catalytic subunit of PKA or the cAMP-analogue Sp-5,6-DCl-cBIMPS between WKY and SHR, despite the differences in the AC-pathway efficacy in these rat strains. Thus, our data suggest that the difference in the iloprost-activated BK current between WKY and SHR does not appear to be due to a difference in the efficacy of the downstream part of the G-protein/AC/BK channel pathway.

This study has its limitations. Strictly speaking, the results only apply to the rat tail artery. It is well established that signaling mechanisms vary in different arteries. Moreover, as mentioned above, even with respect to the G-protein/AC/BK channel signaling pathway, vessel specific differences in hypertension-induced changes were found, e.g., in the functional activity of the G-protein/AC pathway [12] or in BK channel calcium-sensitivity [20,26,27,28,30,36,37]. Thus, our findings cannot simply be transferred to other vascular beds but can be used to develop hypotheses for further studies. Furthermore, the presented study provides new data describing the direct targeting of the BK channel by the G-protein/AC pathway at the cellular level in WKY and SHR. However, this pathway also affects components that regulate the local activator calcium for the BK channel which have been shown to be affected differently in SHR [27,42,43,44,45,46]. Moreover, one study reported that a reduced function of the G-protein/AC pathway in intact arteries in SHR was compensated by an upregulation of BK channels [72]. Thus, due to the complexity of the G-protein/AC/BK channel pathway, the results presented here cannot be used to predict potential functional differences of this pathway in intact arteries of WKY and SHR. However, they serve as a good basis for studies that want to take the next logical step to extend the understanding of the functional activity of the G-protein/AC/BK channel pathway in SHR from the cellular to the organ level.

In summary, our study shows at the cellular level that BK channels, the targets of the G-protein/AC signaling pathway, have similar properties in rat tail arteries in WKY and SHR. Further, BK currents in intact cells of WKY and SHR were similar when the local activator calcium for the channels was strongly buffered. Moreover, downstream activation of the G-protein/AC pathway by direct application of PKA and by PKA activation resulted in a similar increase in BK currents. In contrast, upstream activation of the G-protein/AC pathway by iloprost, which brings to bear the differential efficacy of this pathway, resulted in a smaller increase of the BK current in SHR compared to WKY. Thus, our data suggest that the lower iloprost-induced stimulation of the BK current in freshly isolated rat tail artery smooth muscle cells from SHR compared with WKY is due to the lower efficacy of upstream elements of the G-protein/AC/BK channel pathway.

## 4. Materials and Methods

This study has been conducted in accordance with the US Guide for the Care and Use of Laboratory Animals (Eighth edition, National Academy of Sciences, 2011). Use of laboratory animals has been approved by a local governmental committee on animal welfare.

### 4.1. Dissection of the Vessels

Cell isolation was performed according to the protocol typically used for patch-clamp studies in our laboratory. It has been shown to provide single smooth muscle cells from rat tail arteries with well-preserved PKC, PKG and PKA signaling pathways, as well as long-lasting and stable BK channel activity [47,48,67,73,74]. Briefly, male Wistar–Kyoto rats, 16–25 weeks old, were sacrificed under CO_2_ narcosis by decapitation. The systolic blood pressure of these rats measured with the tail cuff method was different with 215 ± 4 mmHg (*n* = 24) for SHR and 129 ± 2 mmHg (n = 26) for WKY rats (*p* < 0.05; *t*-test). The rat tail was removed and placed into a low calcium physiological salt solution (PSS) containing (in mmol/L): 145 NaCl, 4.5 KCl, 1.2 NaH_2_PO_4_, 1.0 MgSO_4_, 0.1 CaCl_2_, 0.025 EDTA (ethylenediaminetetraacetic acid), 5 HEPES at pH 7.3 at 4 °C. Then, tail arteries were dissected free and cleaned of connective tissue.

### 4.2. Cell Isolation

After dissection, a 1–1.5 cm long piece of a tail artery was placed into a microtube containing 1 mL of an enzyme solution for overnight storage at 4 °C. The enzyme solution contained (in mmol/L): 110 NaCl, 5 KCl, 0.16 CaCl_2_, 2 MgCl_2_, 10 NaHEPES, 10 NaHCO_3_, 0.5 KH_2_PO_4_, 0.5 NaH_2_PO_4_, 10 glucose, 0.49 EDTA, and 10 taurine at pH 7.0, as well as 1.5 mg/mL papain, 1.6 mg/mL albumin and 0.4 mg/mL DL-dithiothreitol. On the next day the microtube with the vessel was incubated for 5–10 min at 37 °C. Single cells were released by trituration with a polyethylene pipette into the experimental bath solution. The experimental bath solution contained: in whole cell experiments (in mmol/L) 135 NaCl, 6 KCl, 0.1 CaCl_2_, 1 MgCl_2_, 3 EGTA (purity 96%), and 10 HEPES at pH 7.4 with a calculated free calcium concentration <10^−7^ mol/L and in inside-out experiments (in mmol/L) 140 KCl, 5 NaCl, 3 EGTA (ethylene-glycol-bis(beta-amino-ethyl-ether)N,N,N’,N’- tetra-acetic acid) (purity 96%), 3 HEDTA (N-hydroxyethyl-ethylene-diaminetriacetic acid) (purity 98%), an appropriate amount of MgCl_2_ to obtain 1 mmol/L free magnesium, an appropriate amount of CaCl_2_ to get different free calcium concentrations, and 10 HEPES at pH 7.4. For a first estimate of the free calcium concentrations, solution composition was calculated with the following apparent reaction constants at pH 7.4: log K_CaEGTA_ = 7.17, log K_MgEGTA_ = 1.93, log K_CaHEDTA_ = 5.67, log K_MgHEDTA_ = 4.37 [75]. Subsequently, the exact free calcium concentration in all solutions was measured with a calcium electrode (KWIKCAL; World Precision Instruments, USA) calibrated with potassium-based standard solutions (CALBUF-2; World Precision Instruments, USA). The pipette solution contained in whole-cell experiments: (in mmol/L): 102 KCl, 10 NaCl, 1 CaCl_2_, 1 MgCl_2_, 0.1 MgATP, 10 EGTA (purity 96%), 10 HEPES at pH 7.4 with a calculated free calcium concentration <10^−8^ mol/L and in inside-out experiments (in mmol/L): 130 NaCl, 15 KCl, 1 MgCl_2_, 3 EGTA (purity 96%), 10 HEPES and an appropriate amount of CaCl_2_ to obtain a free calcium concentration of 3 × 10^−7^ mol/L at pH 7.4.

### 4.3. Patch-Clamp Recording

All experiments were carried out, as described previously [74], at room temperature (22–24 °C). Patch pipettes were prepared from borosilicate glass (WP Instruments, Berlin, Germany), pulled in two stages on a P-30 puller (Sutter Instruments, Novato, CA, USA) and fire polished. They had resistances in the range of 1–5 Mohm. The recordings were made with an Axopatch 200 amplifier (Axon Instruments, Burlingame, CA, USA) with the whole-cell and the inside-out patch-clamp configurations. Single channel data were stored on a DTR-1800 data recorder (Biologic, Seyssinet-Pariset, France) and replayed for analysis, where they were filtered at 1 kHz with use of an eight-pole Bessel filter (model 902, Frequency Devices, Ottawa, IL, USA) and digitized at 5 kHz. Then, they were analyzed off-line with the software package ASCD (G. Droogmans, Lab. Fysiologie, KU Leuven, Belgium). The single channel amplitudes were determined by fitting Gaussian distributions to the amplitude histograms of the closed and the open state, respectively. The activity of the channel in a patch was determined as NPo, where Po is the open probability of one channel and N is the number of channels in the patch. NPo was calculated as the sum of the times finding 1, 2, 3, …, N simultaneously open channels multiplied by the number of open channels and divided by the registration time. The registration time was 30–180 s depending on the level of activity of the channel. N was determined at the end of each experiment with use of a solution with a high calcium concentration and Po was subsequently calculated. All potentials are expressed as membrane potentials.

Multi-channel patches were obtained in most cases. Increasing the patch pipette resistance in order to reduce patch membrane area resulted in vesicle formation during excision, which were resistant to usual rupture procedures. Therefore, multi-channel patches were used and the number of channels in the patch was obtained by applying a high calcium solution at the end of each experiment. Experiments were conducted only on patches with a stable channel activity. In order to obtain channel recordings as close as possible resembling the conditions in an intact cell, immediately after attaining the inside-out configuration, the registration of channel activity was started and lasted 30–180 s, the time necessary to acquire a representative activity at different activity levels of the BK channel. Directly after the end of this period, a high calcium solution was applied and the experiment was stopped.

In whole-cell experiments, stimulation of currents with pulse and ramp protocols, data sampling at a rate of 1 kHz for ion currents and 50 kHz for cell capacitance and series resistance determination, as well as data analysis were all carried out with the software package ISO2 (MFK, Frankfurt, Germany). Experiments were performed only on cells with essentially no leak. Series resistance compensation was not employed because often cells were lost even at slight overcompensation. Instead, series resistance was measured every 2 min. during the course of the experiment. Data were used for analysis if the command voltage error due to series resistance changed was less than 2 mV. Whole-cell currents were elicited every 5 s by either 500 ms step pulses from a holding potential of −40 mV to a test potential of +70 mV or by 1000 ms ramp pulses from −70 mV to +100 mV. Current amplitudes were determined after step pulse stimulation and measured as the mean current of the last 100 ms of the average of 3 consecutive current traces. The BK current was defined as the difference in the net outward current amplitude under the desired condition, e.g., control or iloprost application, and the current amplitude after application of 100 nmol/L iberiotoxin, the specific inhibitor of BK channels, which was given at the end of each experiment. The correctness of this procedure was proven by the observation, that the iberiotoxin-resistant currents after the application of iloprost, PKA or Sp-5,6-DCl-cBIMPS were not different compared to the currents without application of any substance.

### 4.4. Drugs and Chemicals

Albumin, DL-dithiothreitol, as well as the salts for the solutions were obtained from Sigma (Deisenhofen, Germany). Papain was from Ferak (Berlin, Germany). Iloprost was from Tocris (Wiesbaden, Germany). Iberiotoxin was from Research Biochemicals International (Cologne, Germany). Sp-5,6-DCl-cBIMPS (5,6-Dichloro-1-beta-D-ribofuranosylbenzimidazole-3′,5′-cyclic monophosphothioate) was from Biolog (Bremen, Germany) and PKA was from Promega (Mannheim, Germany).

### 4.5. Statistics

All data are presented as mean ± SEM; n is the number of cells. Statistical analysis was performed using repeated measures analysis of variance, two-way and one-way analysis of variance or unpaired *t*-test as appropriate (SPSS 9.0 for Windows, IBM, Ehningen, Germany).

## Figures and Tables

**Table 1:** Dependence of BK channel activity on membrane potential (MP) in inside-out patches excised from tail artery smooth muscle cells of WKY and SHR at different pCa. The data represent the mean of the ratios of the open probability measured at +20 mV and of the open probability measured at 0 mV obtained from the same patch.

	WKY	SHR	*n*	*p*-Value
pCa 5.9	11.6 ± 2.7	15.8 ± 4.9	7	0.48
pCa 5.5	4.6 ± 1.9	4.3 ± 1.2	4	0.89
pCa 4.9	1.5 ± 0.2	1.4 ± 0.3	5	0.95

## Data Availability

All data generated during this study are available from the corresponding author on reasonable request.
